# The HIV Care Cascade from HIV diagnosis to viral suppression in sub-Saharan Africa: a systematic review and meta-regression analysis protocol

**DOI:** 10.1186/s13643-017-0562-z

**Published:** 2017-08-25

**Authors:** Aysel Gueler, Fiona Vanobberghen, Brian Rice, Matthias Egger, Catrina Mugglin

**Affiliations:** 1 Institute of Social and Preventive Medicine (ISPM), University of Bern, Finkenhubelweg 11, 3012 Bern, Switzerland; 2 Population Studies Group, Dept of Population Health, Faculty of Epidemiology and Population Health, London School of Hygiene & Tropical Medicine, Keppel Street, London, WC1E 7HT UK; 3 Measurement & Surveillance of HIV Epidemics Consortium, Faculty of Public Health and Policy, London School of Hygiene and Tropical Medicine, 15-17 Tavistock Place, London, WC1H 9SH UK; 4 Centre for Infectious Disease Epidemiology and Research (CIDER), School of Public Health and Family Medicine, Faculty of Health Sciences, University of Cape Town, Observatory 7925, Cape Town, South Africa

**Keywords:** HIV, HIV care cascade, Antiretroviral therapy, HIV diagnosed, Linkage to care, Viral suppression, HIV care continuum, Systematic review, Meta-analysis, Sub-Saharan Africa

## Abstract

**Background:**

In 2014, UNAIDS announced the 90-90-90 treatment targets to curb the HIV epidemic by 2020: 90% of people living with HIV know their HIV status, 90% of people who know their HIV status access treatment and 90% of people on treatment have suppressed viral loads. Monitoring and evaluation are needed to track linkage and retention throughout the continuum of care. We propose a systematic review and meta-regression to identify the different methodological approaches used to define the steps in the HIV care cascade in sub-Saharan Africa (SSA), where most people with HIV live, and to assess the proportion of participants retained at each step.

**Methods:**

We will include cohort and cross-sectional studies published between 2004 and 2016 that report on the HIV care cascade among adults in SSA. The PubMed, Embase and CINAHL databases will be searched. Two reviewers will independently screen titles and abstracts, assess the full texts for eligibility and extract data. Disagreements will be resolved by consensus or consultation with a third reviewer. We will assess the number and proportion of individuals retained in the HIV care cascade from HIV diagnosis to linkage to care, engagement in pre-ART care, initiation of ART, retention on ART, and viral suppression. The data will be analysed using random effects meta-regression analysis. Publication bias will be assessed by funnel plots.

**Discussion:**

This review will contribute to a better understanding of the HIV care cascade in SSA. It will help programs identify gaps and approaches to improve care and treatment for people living with HIV and reduce HIV transmission.

**Systematic review registration:**

PROSPERO CRD42017055863

**Electronic supplementary material:**

The online version of this article (doi:10.1186/s13643-017-0562-z) contains supplementary material, which is available to authorized users.

## Background

**Fig. 1:**
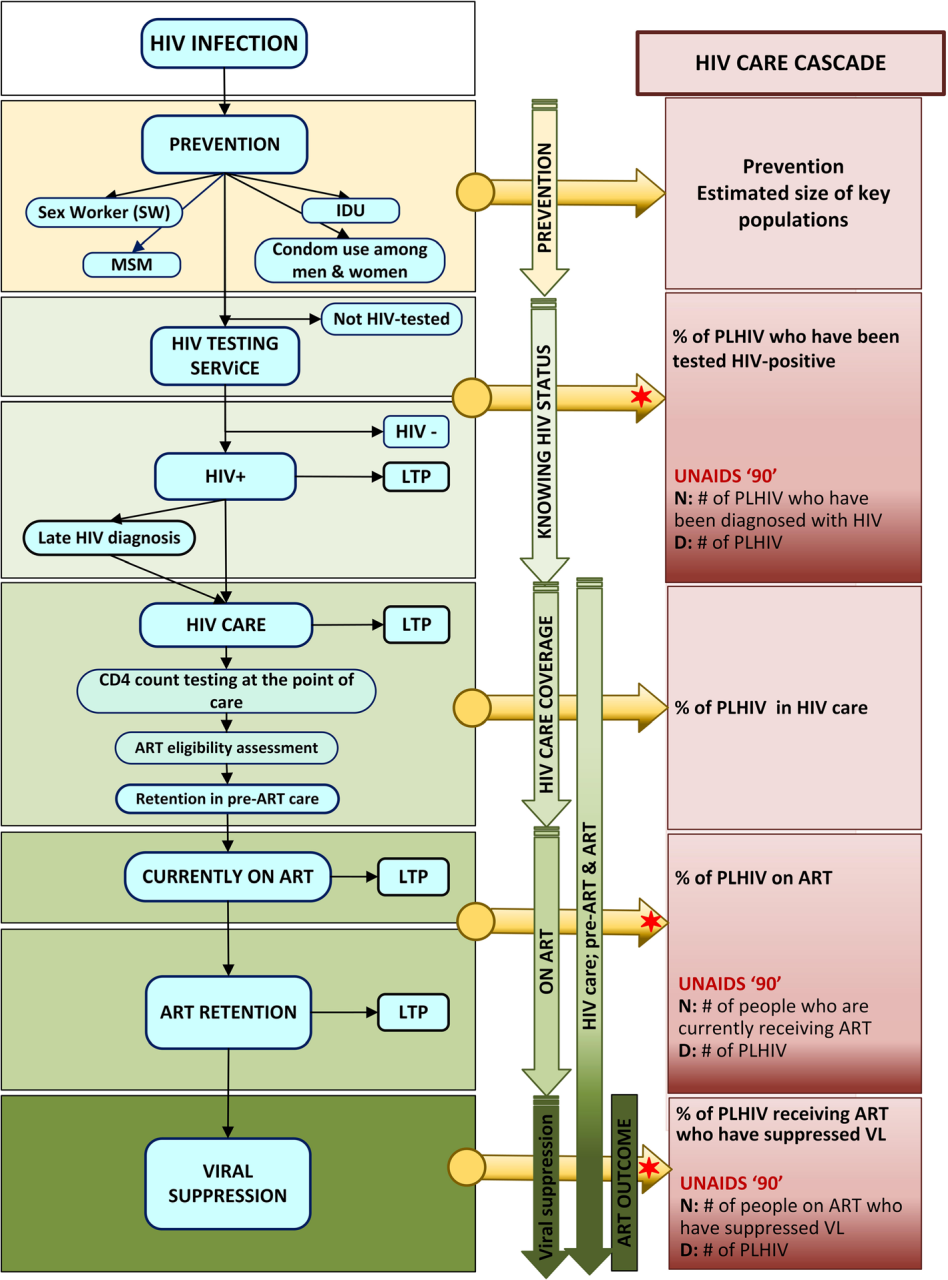
HIV care cascade and assessment of ART eligibility (adapted from WHO guidelines [34]). ART, antiretroviral therapy; IDU, injection drug use; LTP, loss to programme; MSM, men who have sex with men; PLHIV, people living with HIV; VL, viral load

In 2014, UNAIDS proposed the 90-90-90 Fast-Track treatment targets to curb the HIV/AIDS epidemic. These targets stipulate that, by 2020, 90% of people living with HIV worldwide should know their diagnosis, 90% of these people should be on antiretroviral therapy (ART), and 90% of these persons (i.e., 73% of all people living with HIV) should be virally suppressed [1–3]. The HIV care cascade, also known as the HIV care continuum, outlines the sequential steps of HIV care from initial diagnosis to the goal of viral suppression [4, 5] (Fig. 1). To achieve universal access to HIV care and treatment with viral suppression, each HIV-positive individual must progress along this cascade. HIV testing services [6, 7], earlier diagnosis [8, 9], linkage and retention in care [10–12] and earlier ART initiation [13–16] are key components to achieve the 90-90-90 goals [17].

The cascade can be used to evaluate HIV programme performance and serve as a monitoring tool to identify gaps and opportunities for specific interventions to improve outcomes [18]. In sub-Saharan Africa (SSA) and elsewhere, significant gaps remain [19–21], and retention along the HIV care cascade remains a major problem [22–27]. However, studies analysing the HIV care cascade use different methodologies and calculations to construct the cascade. For each element in the cascade, either measured or estimated, definitions vary and methods are often not well described [5]. The use of different measures, for example how retention in HIV care is defined or the threshold for viral suppression, to estimate the cascade stages may affect the results [28]. It is therefore difficult to compare published cascades across regions and calendar periods.

Few studies have evaluated the methods used to define the HIV care cascade in low- and middle-income countries, or in SSA [18]. A previous systematic review from 2014 looking at the whole cascade from HIV testing to viral suppression failed to identify any eligible studies from low-income countries [29]. A more recent review evaluated the cascade based on national estimates but did not include smaller in-depth studies [30]. In SSA, surveys conducted between 2007 and 2011 found that 36% of people in the region had never been tested for HIV [31], and less than 50% of HIV-positive persons knew their HIV status [20]. Focusing on SSA, we aim to perform a systematic review and meta-regression analysis to assess the different methodological approaches used to define the steps in the HIV care cascade and to estimate the numbers or proportion of people retained in each cascade step.

## Methods

This systematic review protocol was written following the Preferred Reporting Items for Systematic Review and Meta-analysis Protocols (PRISMA-P) guidelines [32]. The PRISMA-P checklist can be found as Additional file 1.

### Aims

The first aim of this systematic review is to describe the different concepts and methodologies used in the published literature to define the steps in the HIV care cascade in SSA. The second aim is to obtain comparable measures of the number or proportion of people retained in the different steps of the HIV care cascade, based on the findings from the first aim.

### Eligibility criteria

#### Study designs and setting

We will include cohort and cross-sectional studies with data collected between January 2004 and March 2016 in any country in SSA (Table 1). Studies that examine two or more of the following cascade elements will be included: the number or proportion of persons diagnosed with HIV (first UNAIDS 90-90-90 treatment target), linkage of HIV-positive persons to pre-ART care, retention in pre-ART care, initiating ART, remaining on ART (second UNAIDS 90-90-90 treatment target), and virological suppression among persons on ART (third UNAIDS 90-90-90 treatment target).

**Table 1:** Selection criteria

Criteria	Variables
Inclusion criteria	
	HIV-positive people aged 15 years and older in sub-Saharan Africa
	Study period after 01 January 2004 (ART initiation after 01 January 2004)
	General population
	Reporting on a cascade with two or more elements (HIV diagnosed, linked to care, retained in care, on ART, virally suppressed)
	Observational studies: cohort or cross-sectional studies
	Published in English, French or Spanish
Exclusion criteria	
*Publication type*	
	Narrative reviews
	Commentaries, editorials or letters
	Conference abstracts
*Study design*	
	Randomized controlled trials
	Qualitative studies
	Case series
	Simulations or modelling studies
	Unclear study design
*Study population*	
	Populations outside sub-Saharan Africa
	Children
	Specific sub-populations (for example patients with tuberculosis, or pregnant women)

#### Population

We will include studies of the general population living with HIV. This will encompass male and female participants living with HIV-1 aged 15 years or older. We will exclude studies of patients with specific co-morbidities such as tuberculosis or opportunistic infections. Studies in patients with HIV-2 will also be excluded. If the HIV type is not specified, HIV-1 will be assumed. We will exclude studies of prevention of mother-to-child transmission (PMTCT) as the PMTCT care cascade differs substantially from the general HIV care cascade [33, 34]. We will also exclude intervention studies that examine the effectiveness of measures to improve the HIV care continuum.

#### Outcomes

##### Primary outcomes

The primary outcome is the concept, methodology and definition used to identify a reported stage of the cascade. We will describe these narratively and classify studies into suitable groups.

##### Secondary outcomes

The secondary outcomes include the number or proportion of persons retained at each cascade step (see also Fig. 1 and Additional file 2 ):

- HIV diagnosis (UNAIDS 90-90-90 treatment target)
- Linkage to pre-ART care
- Retention in pre-ART care
- On ART (UNAIDS 90-90-90 treatment target) with
  - Start of ART
  - Retention on ART
- Suppression of viral load (UNAIDS 90-90-90 treatment target)

### Information sources

#### Electronic databases

We have conducted a comprehensive literature search with the help of two librarians with expertise in systematic reviews. We restricted the search to articles published in English, French and Spanish between January 2004 and March 2016, since the scale-up of ART in SSA started around 2004 [35, 36]. We performed the searches in PubMed, Embase and CINAHL.

#### Search strategy

The Medical Subject Headings (MeSH terms) for HIV and AIDS and key terms ‘cascade’, ‘continuum’, ‘linkage to care’, ‘retention in care’ and ‘ART initiation’ were cross-referenced with terms associated with 62 African countries (Additional file 3 shows the detailed search strategy). We will update the search prior to publication to include any additional eligible papers published after March 2016.

### Study records

#### Data management

All records from our PubMed, Embase and CINAHL searches will be combined, uploaded into the reference management software Mendeley (version 1.15.3) and de-duplicated. We will use Microsoft Excel (version 2016 for Windows, Microsoft Corp., Redmond, WA, USA) to record outcomes of the selection process.

#### Selection of eligible studies

Two reviewers will independently screen studies in two stages: title/abstract screening, followed by full text screening. A checklist with the eligibility criteria will be developed and pilot-tested on a random sample of 20 studies. Titles and abstracts will then be reviewed against the eligibility criteria by AG and FV. We will obtain full texts of all potentially eligible articles. Two reviewers will independently apply inclusion criteria (Additional file 4) to the full texts. At both screening steps, we will resolve disagreements by consensus, if necessary through discussion with a third reviewer (CM or ME). We will record all discrepancies on Excel spreadsheets, with reasons for inclusion or exclusion. The PRISMA study flow diagram will reflect this process and detail the reasons for exclusion of studies.

#### Data collection

We will develop a data extraction sheet to guide data collection. This sheet will direct collection of the definition and methods for each step of the cascade, the results of estimations or calculations and sources of data. The sheet will be pilot-tested by two reviewers (AG, CM) on a random sample of 10 articles and revised as needed. Two reviewers will independently read each article and extract the relevant data. Both sets of data will be entered into Epidata version 3.1 (EpiData Association, Denmark). Any discrepancies in the extracted data will be resolved by consensus, in discussion with a third reviewer (CM or ME) if necessary. We will contact study authors to resolve any information that is not clear.

#### Data items

The data items for extraction will be informed by items reported in the PRISMA statement [37–39]. We will extract the following from included studies:

- Study characteristics (authors, year of publication, study period, study population, study design, aim of study, geographic location, duration of follow-up, key finding);
- Study setting (location and type of facilities);
- Characteristics of study populations (sample size, age, sex, marital status, weight, educational level, employment status, enrolment period, inclusion and exclusion criteria);
- Definitions and methods used to construct cascade;
- The number or proportion of persons diagnosed with HIV (90-90-90 treatment target), linkage to pre-ART care, retention in pre-ART care, on ART (90-90-90 treatment target), virological suppression (90-90-90 treatment target);
- Clinical and laboratory data (CD4 cell counts, viral load, ART regimen, medical circumcision (only men), TB status, other co-infections and comorbidities).

#### Data synthesis

The main characteristics of included studies will first be narratively synthesized. Summary statistics will be used to describe study characteristics, including means (standard deviations) or medians (interquartile ranges), and frequencies (percentages). For each step of the cascade, we will calculate proportions with exact binomial 95% confidence intervals (95% CI) and present these in forest plots. We will calculate the between-study variance (tau-squared) and *p* values from tests of between-study heterogeneity. We expect substantial between-study heterogeneity, and the focus of the subsequent analyses will therefore be on the identification and exploration of sources of heterogeneity. We will explore associations between proportions at each step and country, setting (e.g. urban, periurban, rural; health care level, public or private setting) and study characteristics (e.g. study size, sampling frame) using random intercept logistic meta-regression (Binomial-Normal) models. These models avoid the biases that arise when Normal-Normal models (which model the within study variability via normal approximations) are applied to logit or arcsine-square root transformed proportions [40, 41]. Where appropriate, we will use the same models to calculate combined estimates of proportions. All analyses will be done in R version 3.2.3 (R Foundation, Vienna, Austria).

#### Dealing with missing data

If data are missing in key variables, we will contact the study authors for clarification. A description of missing data will be provided for each study, and we will discuss the possible implications of missing data.

#### Risk of bias in included studies

Two pairs of reviewers (AG and CM) will assess included studies using ROBINS-I, a tool for assessing risk of bias in nonrandomized studies of interventions for observational studies [42]. The tool will be adapted to the context of this systematic review, and to cross-sectional studies. ROBINS-I contains 34 questions from seven different bias domains. For each study, relevant domains of risk of bias will be graded as low, moderate, serious, critical or no information for risk of bias [43]. Publication bias will be assessed by visually inspecting funnel plot asymmetry and by including study size in the logistic model. The quality assessment will be cross-checked, and any disagreement will be resolved within the review team.

## Discussion

This systematic review and meta-analysis will contribute to a better understanding of the different methodological approaches used in sub-Saharan African countries to define the steps in the HIV care cascade and to estimate the numbers or percentages of people retained at each step of the cascade. We will identify gaps in the cascade and areas for further research. Our results will be useful for the design of strategies for improving the care and treatment of people living with HIV and for reducing HIV transmission. This review will thus be highly relevant to inform health systems interventions and HIV prevention and treatment strategies in sub-Saharan Africa, and low- and middle-income countries in general.

## Additional files

Additional file 1: PRISMA-P 2015 Checklist. [17 items]. (DOCX 33 kb)

Additional file 2: Complete cascade including all steps. A) HIV care cascade as defined by the WHO, B) Assessment of ART eligibility within the HIV care cascade. ART, antiretroviral therapy; D, denominator; LTP*, loss to programme (pre-ART); LTP**, loss to programme (post-ART); N, nominator; PLD, population level denominator; PBD, programme-based denominator. (JPG 5616 kb)

Additional file 3: Search strategy (PubMed, Embase, and CINAHL). (PDF 79 kb)

Additional file 4: Study eligibility form. (PDF 114 kb)

## Abbreviations

AIDS: Acquired immunodeficiency syndrome
ART: Antiretroviral therapy
CI: Confidence interval
GRADE: Grading of Recommendations Assessment, Development and Evaluation
HBV: Hepatitis B virus
HCV: Hepatitis C virus
HIV: Human immunodeficiency virus
LMIC: Low- and middle-income countries
LTFU: Loss to follow-up
MeSH: Medical subject headings
PMTCT: Prevention of mother-to-child transmission
PRISMA-P: Preferred Reporting Items for Systematic Review and Meta-analysis Protocols
PROSPERO: International Prospective Register of Systematic Reviews
ROBINS-I: Risk Of Bias In Non-randomized Studies - of Interventions
SSA: Sub-Saharan Africa
TB: Tuberculosis
UNAIDS: Joint United Nations Programme on HIV/AIDS
WHO: World Health Organization
