# Supplementary material for: The HIV Care Cascade from HIV diagnosis to viral suppression in sub-Saharan Africa: a systematic review and meta-regression analysis protocol
Source: Syst Rev. 2017 Aug 25;6:172. doi: 10.1186/s13643-017-0562-z (PMC5574086; doi:10.1186/s13643-017-0562-z)
Supplement: Supplementary file 3 — Search strategy (PubMed, Embase, and CINAHL). (PDF 79 kb) [file 13643_2017_562_MOESM3_ESM.pdf]

Database and date	Query
**PubMed**<br><br>**(March 16, 2016)**	(((HIV Infections[MeSH] OR HIV[MeSH] OR hiv[tw] OR hiv-1*[tw] OR hiv-2*[tw] OR hiv1[tw] OR hiv2[tw] OR hiv infect*[tw] OR human immunodeficiency virus[tw] OR human immunedeficiency virus[tw] OR human immuno-deficiency virus[tw] OR human immune-deficiency virus[tw] OR ((human immun*) AND (deficiency virus[tw])) OR acquired immunodeficiency syndrome[tw] OR acquired immunedeficiency syndrome[tw] OR acquired immuno-deficiency syndrome[tw] OR acquired immune-deficiency syndrome[tw] OR ((acquired immun*) AND (deficiency syndrome[tw])) OR "sexually transmitted diseases, viral"[MeSH]) OR (Antiretroviral Therapy, Highly Active[MeSH] OR Anti-Retroviral Agents[MeSH] OR Antiviral Agents[MeSH:noexp] OR ((anti) AND (hiv[tw])) OR antiretroviral*[tw] OR ((anti) AND (retroviral*[tw])) OR HAART[tw] OR cART[tw] OR ((anti) AND (acquired immunodeficiency[tw])) OR ((anti) AND (acquired immunedeficiency[tw])) OR ((anti) AND (acquired immuno-deficiency[tw])) OR ((anti) AND (acquired immune-deficiency[tw])) OR ((anti) AND (acquired immun*) AND (deficiency[tw])))) AND (cascade[Title/Abstract] OR continuum[Title/Abstract] OR "linkage to care"[Title/Abstract] OR "linkage to HIV care"[Title/Abstract] OR "retention in care"[Title/Abstract] OR "HIV treatment access"[Title/Abstract] OR "ART initiation"[Title/Abstract])) AND ("Africa"[Mesh] OR africa[Title/Abstract] OR maghreb*[Title/Abstract] OR maghrib*[Title/Abstract] OR Sahara*[Title/Abstract] OR sub-saharan*[Title/Abstract] OR Benin[Title/Abstract] OR Botswana[Title/Abstract] OR Burkina Faso[Title/Abstract] OR Burundi[Title/Abstract] OR "British Indian Ocean Territory"[Title/Abstract] OR Cape Verde[Title/Abstract] OR Canary Islands[Title/Abstract] OR Cameroon[Title/Abstract] OR Ceuta[Title/Abstract] OR Chad[Title/Abstract] OR Comoros[Title/Abstract] OR Cote d'Ivoire[Title/Abstract] OR Egypt[Title/Abstract] OR Eritrea[Title/Abstract] OR Ethiopia[Title/Abstract] OR Equatorial Guinea[Title/Abstract] OR Gabon[Title/Abstract] OR Gambia[Title/Abstract] OR Ghana[Title/Abstract] OR Guinea[Title/Abstract] OR Guinea-Bissau[Title/Abstract] OR Djibouti[Title/Abstract] OR Ivory Coast[Title/Abstract] OR Kenya[Title/Abstract] OR Lesotho[Title/Abstract] OR Liberia[Title/Abstract] OR Libya[Title/Abstract] OR Madeira[Title/Abstract] OR Melilla[Title/Abstract] OR Morocco[Title/Abstract] OR Madagascar[Title/Abstract] OR Malawi[Title/Abstract] OR Mauritius[Title/Abstract] OR Mayotte[Title/Abstract] OR Mozambique[Title/Abstract] OR Mali[Title/Abstract] OR Mauritania[Title/Abstract] OR Namibia[Title/Abstract] OR Niger[Title/Abstract] OR Nigeria[Title/Abstract] OR Reunion[Title/Abstract] OR Rwanda[Title/Abstract] OR Republic of the Congo[Title/Abstract] OR Democratic Republic of the Congo[Title/Abstract] OR Seychelles[Title/Abstract] OR South Africa[Title/Abstract] OR Somalia[Title/Abstract] OR Senegal[Title/Abstract] OR Sierra Leone[Title/Abstract] OR Saint Helena[Title/Abstract] OR Sudan[Title/Abstract] OR South Sudan[Title/Abstract] OR Swaziland[Title/Abstract] OR Sao Tome and Principe[Title/Abstract] OR Tanzania[Title/Abstract] OR Togo[Title/Abstract] OR Tunisia[Title/Abstract] OR Uganda[Title/Abstract] OR Western Sahara[Title/Abstract] OR Zambia[Title/Abstract] OR Zimbabwe[Title/Abstract]) Filters: Publication date from 2004/01/01 to 2016/12/31
**Embase**<br><br>**OvidSP**<br><br>**March 17, 2016**	1. exp antiretrovirus agent/<br>2. exp highly active antiretroviral therapy/<br>3. exp antivirus agent/<br>4. ((anti and hiv) or antiretroviral* or (anti and retroviral*) or HAART or (anti and acquired immunodeficiency) or (anti and acquired immunedeficiency) or (anti and acquired immuno-deficiency) or (anti and acquired immune* and deficiency)).tw.<br>5. or/1-4<br>6. exp Human immunodeficiency virus infection/<br>7. (hiv or hiv?1 or hiv?2 or human immun#deficiency virus or human immun#-deficiency virus or (human immun# adj3 deficiency virus) or acquired immun#deficiency syndrome or acquired immun#-deficiency syndrome or (acquired immun# adj3 deficiency syndrome)).tw.<br>8. or/6-7<br>9. (cascade or continuum or "linkage to care" or "linkage to HIV care" or "retention in care" or "HIV treatment access" or "ART initiation").tw.<br>10. 5 or 8<br>11. 9 and 10<br>12. exp Africa/<br>13. (africa or Maghreb* or maghrib* or Sahara* or sub-saharan* or Benin or Botswana or Burkina Faso or Burundi or British Indian Ocean Territory or Cape Verde or Canary Islands or Cameroon or Ceuta or Chad or Comoros or Cote d'Ivoire or Egypt or Eritrea or Ethiopia or Equatorial Guinea or Gabon or Gambia or Ghana or Guinea or Guinea-Bissau or Djibouti or Ivory Coast or Kenya or Lesotho or Liberia or Libya or Madeira or Melilla or Morocco or Madagascar or Malawi or Mauritius or Mayotte or Mozambique or Mali or Mauritania

	or Namibia or Niger or Nigeria or Reunion or Rwanda or Republic of the Congo or Democratic Republic of the Congo or Seychelles or South Africa or Somalia or Senegal or Sierra Leone or Saint Helena or Sudan or South Sudan or Swaziland).tw.<br>14. ("Sao Tome and Principe" or Tanzania or Togo or Tunisia or Uganda or Western Sahara or Zambia or Zimbabwe).tw.<br>15. 12 or 13 or 14<br>16. 11 and 15<br>17. limit 16 to yr="2004 - 2016"
**CINAHL**<br><br>**March 21, 2016**	S1 (MH "Antiretroviral Therapy, Highly Active")<br>S2 (MH "Anti-Retroviral Agents+")<br><br>S3 (MH "Antiviral Agents")<br>S4 TI ((anti and hiv) or antiretroviral* or (anti and retroviral*) or HAART or cART or (anti and acquired immunodeficiency) or (anti and acquired immunedeficiency) or (anti and acquired immuno-deficiency) or (anti and acquired immun* and deficiency)) OR AB ((anti and hiv) or antiretroviral* or (anti and retroviral*) or HAART or cART or (anti and acquired immunodeficiency) or (anti and acquired immunedeficiency) or (anti and acquired immuno-deficiency) or (anti and acquired immun* and deficiency)<br>S5 S1 OR S2 OR S3 OR S4<br>S6 (MH "HIV Infections+")<br>S7 (MH "Human Immunodeficiency Virus+")<br>S8 TI (hiv or hiv?1 or hiv?2 or human immun#deficiency virus or human immun#-deficiency virus or (human immun# N3 deficiency virus) or acquired immun#deficiency syndrome or acquired immun#-deficiency syndrome or (acquired immun# N3 deficiency syndrome)) OR AB (hiv or hiv?1 or hiv?2 or human immun#deficiency virus or human immun#-deficiency virus or (human immun# N3 deficiency virus) or acquired immun#deficiency syndrome or acquired immun#-deficiency syndrome or (acquired immun# N3 deficiency syndrome)<br>S9 (MH "Sexually Transmitted Diseases, Viral+")<br>S10 S6 OR S7 OR S8 OR S9<br>S11 TI (cascade or continuum or "linkage to care" or "linkage to HIV care" or "retention in care" or "HIV treatment access" or "ART initiation") OR AB (cascade or continuum or "linkage to care" or "linkage to HIV care" or "retention in care" or "HIV treatment access" or "ART initiation")<br>S12 S5 OR S10<br>S13 S11 AND S12<br>S14 (MH "Africa+")<br>S15 TI (africa or Maghreb* or maghrib* or Sahara* or sub-saharan* or Benin or Botswana or Burkina Faso or Burundi or British Indian Ocean Territory or Cape Verde or Canary Islands or Cameroon or Ceuta or Chad or Comoros or Cote d'Ivoire or Egypt or Eritrea or Ethiopia or Equatorial Guinea or Gabon or Gambia or Ghana or Guinea or Guinea-Bissau or Djibouti or Ivory Coast or Kenya or Lesotho or Liberia or Libya or Madeira or Melilla or Morocco or Madagascar or Malawi or Mauritius or Mayotte or Mozambique or Mali or Mauritania or Namibia or Niger or Nigeria or Reunion or Rwanda or Republic of the Congo or Democratic Republic of the Congo or Seychelles or South Africa or Somalia or Senegal or Sierra Leone or Saint Helena or Sudan or South Sudan or Swaziland) OR AB (africa or Maghreb* or maghrib* or Sahara* or sub-saharan* or Benin or Botswana or Burkina Faso or Burundi or British Indian Ocean Territory or Cape Verde or Canary Islands or Cameroon or Ceuta or Chad or Comoros or Cote d'Ivoire or Egypt or Eritrea or Ethiopia or Equatorial Guinea or Gabon or Gambia or Ghana or Guinea or Guinea-Bissau or Djibouti or Ivory Coast or Kenya or Lesotho or Liberia or Libya or Madeira or Melilla or Morocco or Madagascar or Malawi or Mauritius or Mayotte or Mozambique or Mali or Mauritania or Namibia or Niger or Nigeria or Reunion or Rwanda or Republic of the Congo or Democratic Republic of the Congo or Seychelles or South Africa or Somalia or Senegal or Sierra Leone or Saint Helena or Sudan or South Sudan or Swaziland)<br>S16 TI ("Sao Tome and Principe" or Tanzania or Togo or Tunisia or Uganda or Western Sahara or Zambia or Zimbabwe) OR AB ("Sao Tome and Principe" or Tanzania or Togo or Tunisia or Uganda or Western Sahara or Zambia or Zimbabwe)<br>S17 S14 OR S15 OR S16<br>S18 S13 AND S17<br>S19 S13 AND S17 Limiters - Published Date: 20040101-20161231
