# Supplementary material for: The HIV Care Cascade from HIV diagnosis to viral suppression in sub-Saharan Africa: a systematic review and meta-regression analysis protocol
Source: Syst Rev. 2017 Aug 25;6:172. doi: 10.1186/s13643-017-0562-z (PMC5574086; doi:10.1186/s13643-017-0562-z)
Supplement: Supplementary file 4 — Study eligibility form. (PDF 114 kb) [file 13643_2017_562_MOESM4_ESM.pdf]

**Study ID**-Number:	**[Study-ID number:** Use the code from the **EXCEL-Sheet**]
**STUDY PERIOD**<br>1. Study period after 01.01.**2004** (ART initiation after 01.01.2004)	☐ YES ⇩ ☐ UNCLEAR ⇩ ☐ NO ⇨ **EXCLUDE**
**PARTICIPANTS**<br>2. HIV-1-infected patients aged 15 years or older (non HIV-2)	☐ YES ⇩ ☐ UNCLEAR ⇩ ☐ NO ⇨ **EXCLUDE**
**SETTING**<br>3. People living with HIV in sub-Saharan AFRICA	☐ YES ⇩ ☐ UNCLEAR ⇩ ☐ NO ⇨ **EXCLUDE**
4. "General population"<br>(not a specific sub population is studied such as TB infected, PMTCT, second-line ART only, opportunistic infection, only patients with cancer, drug resistance, malaria, serodiscordant couples)	☐ YES ⇩ ☐ UNCLEAR ⇩ ☐ NO ⇨ **EXCLUDE**
**OUTCOMES**<br>5. At least TWO elements of the following HIV Care Cascade were measured:<br>a. HIV diagnosed ☐<br>b. Linked to care ☐<br>c. Engaged or retained in care ☐<br>d. On ART ☐<br>e. Virally suppressed ☐	☐ YES ⇩ ☐ UNCLEAR ⇩ ☐ NO ⇨ **EXCLUDE**
6. At least ONE element of the following HIV Care Cascade was measured (UNAIDS):<br>a. Proportion HIV diagnosed ☐<br>b. Proportion on ART ☐<br>c. Proportion virally suppressed ☐	☐ YES ⇩ ☐ UNCLEAR ⇩ ☐ NO ⇨ **EXCLUDE**
**STUDY DESIGNS**<br>7. Study design:<br>a. Cohort study ☐<br>b. Cross sectional study ☐	☐ YES ⇩ ☐ UNCLEAR ⇩ ☐ NO ⇨ **EXCLUDE**
**FINAL DECISION**<br>1x EXCLUDE = EXCLUDE<br>1x UNCLEAR = UNCLEAR	☐ INCLUDE ☐ UNCLEAR ☐ **EXCLUDE**
**NOTES**<br>☐ yes ☐ no<br>**Please do not forgot to add the publication type (journal article, letter, conference paper, or other)*	Publication type:<br>☐ Journal article ☐ Editorial/letter ☐ Conference paper ☐ Other
